# Supplementary material for: Research on the internal flow and macroscopic characteristics of a diesel fuel injection process
Source: PLoS One. 2021 Sep 27;16(9):e0255874. doi: 10.1371/journal.pone.0255874 (PMC8476005; doi:10.1371/journal.pone.0255874)
Supplement: Attachment — Submitted filename: Response to Reviewers.docx [file pone.0255874.s001.docx]

**Response to Reviewers**

Dear Editor-in-chief:

We have considered the suggestions of the reviewers carefully and re-submitted the manuscript according to your kind advice. Thank you for your letter and for the reviewers’ comments concerning our manuscript entitled “Research on the Internal Flow and Macroscopic Characteristics of a Diesel Fuel Injection Process”. Those comments are all valuable and very helpful for revising and improving our paper, as well as the important guiding significance to our researches. We have studied comments carefully and have made correction which we hope meet with approval. The corrections in the paper and the responds to the reviewer’s comments are as following:

**Editor**

1. Please ensure that your manuscript meets PLOS ONE's style requirements, including those for file naming. The PLOS ONE style templates can be found at

<https://journals.plos.org/plosone/s/file?id=wjVg/PLOSOne_formatting_sample_main_body.pdf> and

<https://journals.plos.org/plosone/s/file?id=ba62/PLOSOne_formatting_sample_title_authors_affiliations.pdf>

2. Please amend either the title on the online submission form (via Edit Submission) or the title in the manuscript so that they are identical.

3. Please ensure that you refer to Figure 5 in your text as, if accepted, production will need this reference to link the reader to the figure.

**Response**: Thank you for your comments. I have revised the article as required.

**Reviewer #1**: Paper title: Research on the Internal Flow and Macroscopic Characteristics of a Diesel Fuel Injection Process

The paper studied the internal flow and the macroscopic characteristics of a diesel fuel injection process. The topic is interesting, the results are discussed in detail. However, there are some flaws to be revised, also the organization is not proper. Here are the detailed comments:

1. The organization of paper needs to be adjusted. The authors use both CFD and experimental methods to study the topic, thus the research object (nozzle) as well as the studied conditions (in CFD, it is expressed as boundary conditions, in experiment, it is the experimental conditions, in either way, the detailed conditions should be the same). Consequently, the nozzle geometry (Fig. 1 and Table 1) and the studied conditions (Table 2) should not be placed in CFD part.

**Response**: Thank you for your comments. I adjusted the position of the nozzle geometry and the studied conditions in the paper. For details, please refer to the revision of Chapter 2 and Chapter 3 in the paper.

2. As I have mentioned, the nozzle structure and the studied conditions should be placed in another part. In addition, the CFD results and the experiment results should be clearly stated, rather than be confused with each other.

**Response**：Thank you for your comments. The result of inner flow is obtained through CFD simulation. The macro spray characteristics are obtained through experiments. I have stated these clearly in our revised manuscript.

3. As the most results are obtained from experiment, only the “Internal flow” part uses the CFD results (if I understand correctly), I suggest the “Description of the CFD method” merges with the “Internal flow”. In this case, the readers can clearly know the CFD study is conducted for the internal flow analysis, not the spray analysis. In this case, the detailed CFD setup, eg., govern equations can choose not be listed. If the authors believe the CFD plays as important role, then more detailed CFD setup, eg., govern equations and CFD results validation, needs to be listed.

**Response**：Thank you for your comments. Yes, only the internal flow part uses the CFD results, and the most results are obtained from experiment. I Combine "Description of CFD Method" with "Internal Flow" as you suggest. Therefore, the detailed CFD setup and govern equations were not listed in the manuscript. The simulation results and the experiment results were clearly stated in our revised manuscript.

4. The authors separated the results by the effect of the injection and ambient pressures, in my opinion, it is not a good choice. The injection and ambient pressures work together to effect the orifice discharge coefficient and the spray characteristics. Therefore, the authors are suggested to discuss these two pressures together, especially the discharge coefficient, it is a function of the differential pressure. In addition, the subtitle “Internal flow” is not clear, maybe the “Volume fraction distribution” is better.

**Response**：Thank you for your comments. Firstly, the change of injection pressure and ambient back pressure have different effects on the cavitation and the downstream spray behaviors. In the case of high-pressure injection, as the injection pressure increases, the flow characteristics inside the nozzle will be affected. In the case of a certain fuel injection pressure, the change of the environmental back pressure has a great influence on the fuel spray. In order to explore the influence of the fuel injection pressure and the environmental back pressure on the nozzle internal flow characteristics and the fuel spray characteristics. That's why they were studied separately from each other. Secondary, the discharge coefficient is a function of the differential pressure, when we study the effectsof injection pressure or the back pressure on Cd, we always fix a constant pressure value, and only change the injection pressure or the back pressure.The effect is the same as changing the differential pressure. Meanwhile, the internal flow has been changed with vapor volume fraction distribution.

5. The results should be discussed more depth combined with the spray process and breakup mechanism. In addition, the important conclusions and explanations needs to be supported by the detailed results or the literature references, eg., in the beginning of Page 17 “which indicates that cavitation produces an increase in the spray cone angle, mainly due to broken cavitation bubbles, which induce many micro fluid jets and increase the radial velocity of the jet, thus increasing the spray cone angle, especially during the primary spray process”. How the authors draw the conclusion without any broken cavitation bubbles pictures or any references? There are many sentences not rigorous enough.

**Response**：Thank you for your comments. Based on your opinion, I have carefully revised the results analysis, interpretation and relevant conclusions in the full text.

Combining with the questions you raised on page 17, the spraying process and the crushing mechanism, the results in the paper are analyzed and compared with the conclusions of the references, and the references are marked in the corresponding positions of the article. See the red part in the article.

6. Fig.1 is not clear to show the detailed structure of the nozzle, the authors should considered the section map.

**Response**：Thank you for your comments. Figure 1 is a cross-sectional view of the nozzle needle according to the parts drawing, and the schematic diagram of the nozzle structure has been modified. The enlarged schematic diagram of the nozzle hole has been added and the relevant geometric parameters have been marked. And add Table 1 to supplement the nozzle structure parameters. The relevant content has been revised in the paper and marked in red.

7. In general, the symbols indicating physical quantity symbols are in italics, and the others are in roman. Please check whether all the subscripts and symbols in this paper are used correctly.

**Response**：Thank you for your comments. Sorry for our unclear writing, we have check the symbols and revised in our revised manuscript.

We have studied reviewer’s comments carefully and have made revision which marked in red in the paper. We have tried our best to revise our manuscript according to the comments, and We would like to submit for your kind consideration.We would like to express our great appreciation to you and reviewers for comments on our paper. Looking forward to hearing from you.

Thank you and best regards.

Yours sincerely

Corresponding author

Name :Hua Xia

E-mail: 250930192@qq.com
