# Supplementary material for: Research on the internal flow and macroscopic characteristics of a diesel fuel injection process
Source: PLoS One. 2021 Sep 27;16(9):e0255874. doi: 10.1371/journal.pone.0255874 (PMC8476005; doi:10.1371/journal.pone.0255874)
Supplement: Attachment — Submitted filename: Response to Reviewers 2.docx [file pone.0255874.s002.docx]

**Response to Reviewers**

Dear Editor and reviewers

Thank you for your view letter and the comments concerning our manuscript entitled ‘Research on the Internal Flow and Macroscopic Characteristics of a Diesel Fuel Injection Process’. Those comments are all very valuable and helpful for revising and improving our paper. We have studied comments carefully and have made correction which we hope meet with approval. The corrections in the paper and the responds to the comments are as following:

**Reviewer #1:** The revised paper is largely improved compared with the original version, and the most problems I mentioned have been addressed. I have some comments to further improve the quality of the paper.

1. The subsection title “The boundary conditions” is not proper. As I have mentioned, the boundary conditions are the exclusive expression in CFD simulation, it includes the boundary setup in CFD. Maybe “The groups of operational conditions” is better. In addition, the injection pressure and ambient pressure are strongly suggested to be named with the variable symbol, for example, Pin and P0, as well as in the whole text and the figures. It is more clear and readable.

**Response**: Thank you for your comments. We have revised these in our revised manuscript.$P_{in}$ is the injection pressure, $P_{out}$is the ambient pressure. We have changed the original title to “The groups of operational conditions”.

2. The variable symbols are not rigorous, please check the paper thoroughly to ensure all the subscript and symbol are identical. For example, K-factor in equation (1), it is K or k？In equation (2), P should be italic, the symbol “Cd” in Fig. 6 should be identical with it in equation (2).

**Response**: Thank you for your comments. Sorry for our unclear writing, it is K; We have revised these in our revised manuscript. We have checked the full text to prevent similar errors.

3. The captions of the figure is not clear, they should be self-evidence. The detailed operational conditions of the results should be given clearly. For example, what is the ambient pressure in Fig.10？The detailed information should be given. In addition, Fig. 7 has typographical error. As I have mentioned, the corresponding variable symbols are suggested to add.

**Response**: Thank you for your comments. The ambient pressure in Fig.10 is 5MPa, we have added in the manuscript. We have modified the titles of several figures to make them more accurate.

4. The logical relation and relevance between the experimental and numerical results should be more clearer.

**Response**: Thank you for your comments. The computational results of inner flow in nozzle orifice are used to analyze the mechanism and reason of the difference between the experimental results of spray characteristics. This part was explained in the part of spray macroscopic characteristics.

5. There are still some grammatical or typographical errors, please check it clearly to avoid it.

**Response**: Thank you for your comments. We have revised the grammatical or typographical errors in our revised manuscript ensure that readers can clearly understand the paper.

We have studied reviewer’s comments carefully and we have tried our best to revise our manuscript according to the comments, and the words in red font were the modified content in the revised manuscript. We would like to submit for your kind consideration. We would like to express our great appreciation to you for comments on our paper. Looking forward to hearing from you.

Thank you and best regards.

Yours sincerely

Corresponding author

Name: **Hua Xia**

E-mail: 250930192@qq.com
